# Policy on infant formula industry funding, support or sponsorship of articles submitted for publication

**DOI:** 10.1186/1746-4358-2-5

**Published:** 2007-03-06

**Authors:** Annette Beasley, Lisa H Amir

**Affiliations:** 1 School of Social and Cultural Studies, Victoria University of Wellington, New Zealand; 2 Mother & Child Health Research, La Trobe University, Melbourne, Australia

## Abstract

Despite current scientific evidence that artificial feeding is a harmful practice, unquestioned acceptance of breastfeeding as the normal or "default" method of infant feeding remains elusive in the industrialised world. Throughout the developing world the profound consequences of the aggressive marketing strategies of the infant formula industry since the end of the Second World War is well known. A key objective of the *International Breastfeeding Journal *is to promote breastfeeding through addressing issues that encourage breastfeeding initiation, duration and effective management. Informing this aim is the recognition of artificial feeding as a harmful practice that places infant health at risk. From this perspective it would be unethical for this journal to accept for publication any manuscript that has received funding, sponsorship or any other means of support from infant formula manufacturers. This stance is consistent with the journal's aim of supporting, protecting and promoting breastfeeding. It will also contribute to the promotion of a breastfeeding culture.

## Editorial

Despite current scientific evidence that artificial feeding is a harmful practice [1], unquestioned acceptance of breastfeeding as the normal or "default" method of infant feeding [2] remains elusive throughout the industrialised world. The dominance of an infant formula feeding culture is evident through popular beliefs such as "artificial milk feeds [are]...at least as good for the baby (if not actually better) as breast feeding", and ambivalent views associated with "give it a go" attitudes [3]. This lack of a breastfeeding culture in most industrialised nations is the legacy of decades of commercial marketing of infant formula, often endorsed by medical practices [4].

Throughout the developing world the profound consequences of the aggressive marketing strategies of the infant formula industry particularly since the end of the Second World War is well known. Public awareness of the tragic outcome of the use of infant formula in the developing world led to a consumer boycott of Nestlé products during the late 1970s and early 1980s. Around this time the first of a series of international strategies designed to reduce infant mortality rates through regulating the promotion of artificial baby milk was implemented. In 1981, the World Health Organization (WHO) announced the International Code of Marketing of Breast-milk Substitutes [5]. The aim of the International Code was "to contribute to the provision of safe and adequate nutrition for infants, by the protection and promotion of breast-feeding, and by ensuring the proper use of breast-milk substitutes, when these are necessary, on the basis of adequate information and through appropriate marketing and distribution" [5] (p.8). While a significant move, universal implementation of the International Code has been hampered by the difficulty of enforcing compliance among members of the infant formula industry and within non-signatory nations.

Following the launch of the International Code, the World Health Assembly has issued a succession of policy statements designed to increase global breastfeeding rates. Of particular significance was the 1989 joint WHO/UNICEF statement on the *Protection, Promotion and Support of Breastfeeding: the Ten Steps to Successful Breastfeeding *[6]. This statement aimed "to encourage hospitals and healthcare facilities to adopt practices that fully protect, promote and support breastfeeding". The internationally applicable Baby Friendly Hospital Initiative is currently the central aspect of a global strategy designed to foster the initiation of breastfeeding at the time of birth [7].

Despite several decades of initiatives by organisations such as WHO, UNICEF and other non-government bodies, for example, World Alliance for Breastfeeding Action, progress in fostering a breastfeeding culture among the industrialised nations has been slow. Hampered by the ideology of consumer choice that promotes a woman's right to decide the method of infant feeding that best suits her lifestyle and worldview, the goal of unquestioned acceptance of breastfeeding as the norm remains elusive. Not only does a consumer culture disregard the right of every infant to be breastfed, but it also fosters accusations that promotion of breastfeeding leads to guilt among those unwilling or unable to successfully establish and maintain lactation. Overlooked by such claims is the hegemonic influence of the infant formula industry, promoted and sustained over decades of marketing of artificial baby milk. As Wiessinger points out, the industry's influence has permeated even the language employed by health professionals to encourage women to breastfeed [8]. For example, promotion of the "advantages" of breastfeeding mimics the commercial sales pitch adopted by the formula industry and so endorses the notion of consumer choice. Wiessinger argues that because breastfeeding is the biological norm, breastfed babies should not be perceived as "healthier", nor do they "smell better" than formula-fed infants and so on [8] (p1). Breastfed babies are simply "normal"!

The profound consequences of the harmful impact of formula marketing practices have generated ethical debate over any form of support received from the artificial baby milk industry. In the late 1990s, Lucas argued that "provided . . . researchers declare their funding sources, they should not be censored for industrial collaborative research into child health" [9] (p. 337) because "formula companies provide a critical contribution to infant health care, health education, and high quality research" [9] (p.338). Others strongly disagreed [10-12]. Rundall pointed out that sponsorship is "a payment by a business firm . . . for the purposes of promoting its name, products or services. It is a commercial deal, not a philanthropic gift" [10] (p338). She argued that companies use sponsorship as a marketing strategy to create the image that they are responsible corporate citizens and to link their name to prestigious organisations and doctors [10]. Others mentioned the "inevitability of bias towards formula feeding in those who take money from the industry" [11] p 260.

"Sponsorship bias" in pharmaceutical research has been confirmed; a meta-analysis found industry sponsorship was associated with an odds of 3.6 (95% Confidence Interval [CI] 2.63, 4.91) of a pro-industry conclusion [13]. A recent systematic review of nutrition-related scientific articles focusing on soft drinks, juices and cow milk, examined the relationship between funding source and conclusion [14]. Again, industry funding was found to favour the sponsor's products. The odds of an article reporting a conclusion favourable to the nutrition industry versus an unfavourable conclusion was 7.61 (95% CI 1.27, 45.73) comparing articles with all industry funding to no industry funding [14]. Among the possible ways to reduce bias in nutrition articles, the authors suggested voluntary refusal by scientists to accept industrial support and the implementation of more stringent policies by journals over the publication of industry-sponsored research [14].

The call to eliminate commercial bias from scientific publications on infant nutrition is consistent with the spirit of the International Code of Marketing of Breast Milk Substitutes. In Britain, for example, direct to the public advertising of formula for infants aged under six months is prohibited in compliance with the International Code. Despite this law, formula advertising remains permitted in health professional journals provided information is restricted to "a scientific and factual nature" [15]. In light of the findings of sponsor bias in nutrition articles, the argument that there is a need to disseminate infant feeding information to all parents in the interests of infant well-being becomes highly questionable. Recently, Magda Sachs, one of the members of the *International Breastfeeding Journal's *Editorial Board, challenged the situation arguing, "Milk company advertising has no place in reputable journals", particularly in Britain where it represents attempts by the industry 'to circumvent the [country's] ban on direct advertising to mothers" [15] (p. 714). She also expressed concern that infant formula advertising may "entice [health] professionals to abandon their independence as practitioners" [15] (p. 714). Sachs made the comments in response to the pro-advertising stance of *British Journal of Midwifery *managing director and publisher, Mark Allen. Among other things, Allen advocates the parents' right of choice, and concern that exclusion of infant formula advertising would discriminate against parents who do not breastfeed [16]. Informing Allen's stance is the view that "we are professional publishers, not moral guardians" [16] (p. 715).

The Sachs/Allen debate draws attention to the challenges that need to be overcome if breastfeeding is to be accepted as the unquestioned "default" method of infant feeding. Central to this challenge are the ethics of the industry's funding, sponsorship and support of infant feeding research. Not only does such patronage jeopardise the independence of researchers and health professionals alike but more importantly it promotes and sustains a formula feeding culture.

A key objective of the *International Breastfeeding Journal *is to promote breastfeeding through addressing issues that encourage breastfeeding initiation, duration and effective management. Informing this aim is the recognition of artificial feeding as a harmful practice that places infant health at risk. From this perspective it would be unethical for this journal to accept for publication any manuscript that has received funding, sponsorship or any other means of support from infant formula manufacturers. This stance is consistent with the journal's aim of supporting, protecting and promoting breastfeeding. It will also contribute to the promotion of a breastfeeding culture.

## Competing interests

The authors declare that they have no competing interests.

## Authors' contributions

AB wrote the first draft of the paper. LHA conceived the idea for the editorial and contributed to the writing.
